# Landscapes of HLA Mismatching in Contemporary Unrelated Haematopoietic Cell Transplantation

**DOI:** 10.1111/tan.70637

**Published:** 2026-03-11

**Authors:** Esteban Arrieta‐Bolaños, Edouard F. Bonneville, Pietro Crivello, Tobias Gedde‐Dahl, Régis Peffault de Latour, Urpu Salmenniemi, Nicolaus Kröger, Ibrahim Yakoub‐Agha, Marco Zecca, Goda Choi, Charles Crawley, Eleni Tholouli, Valérie Dubois, Juha Peräsaari, Lotte Wieten, Steven G. E. Marsh, Mats Bengtsson, Jorinde D. Hoogenboom, Jürgen Kuball, Florent Malard, Annalisa Ruggeri, Katharina Fleischhauer

**Affiliations:** ^1^ Institute for Experimental Cellular Therapy University Hospital Essen Essen Germany; ^2^ German Cancer Consortium (DKTK), Partner Site Essen/Düsseldorf Essen Germany; ^3^ Department of Biomedical Data Sciences LUMC Leiden the Netherlands; ^4^ EBMT Leiden Study Unit Leiden the Netherlands; ^5^ Oslo University Hospital, Rikshospitalet Oslo Norway; ^6^ Saint‐Louis Hospital BMT Unit Paris France; ^7^ HUCH Comprehensive Cancer Center Helsinki Finland; ^8^ University Medical Center Hamburg Hamburg Germany; ^9^ CHU de Lille Univ Lille, INSERM U1286 Lille France; ^10^ San Matteo Pavia Transplant Programme Fondazione IRCCS Policlinico San Matteo Pavia Italy; ^11^ University Medical Center Groningen University of Groningen Groningen the Netherlands; ^12^ Addenbrookes Hospital Cambridge Cambridge UK; ^13^ Manchester Royal Infirmary Manchester UK; ^14^ Histocompatibility Laboratory EFS Lyon Lyon France; ^15^ Clinical Laboratory Services, Histocompatibility Testing Finnish Red Cross Blood Service Vantaa Finland; ^16^ Transplantation Immunology Maastricht University Medical Center Maastricht the Netherlands; ^17^ UCL Cancer Institute London UK; ^18^ Department of Immunology, Genetics and Pathology Uppsala University Uppsala Sweden; ^19^ Department of Hematology University Medical Center Utrecht Utrecht the Netherlands; ^20^ Sorbonne Université Centre de Recherche Saint‐Antoine (CRSA) INSERM UMRs938, Service d'Hématologie Clinique et Thérapie Cellulaire, Hôpital Saint‐Antoine AP‐HP Paris France; ^21^ San Raffaele Scientific Institute Hematology and Bone Marrow Transplantation Unit Milan Italy

**Keywords:** alloreactivity, functional matching, histocompatibility, HLA, immunopeptidome, mismatches

## Abstract

Haematopoietic cell transplantation (HCT) with HLA‐mismatched unrelated donors (MMUD) offers access to curative therapy for patients lacking well‐matched donors. Accumulating evidence suggests that functional matching among allele‐mismatched pairs can significantly influence patient outcomes. Therefore, real‐world data on mismatch frequencies in MMUD‐HCT could provide fundamental information for the assessment of patient risks and donor selection strategies. Here, we analysed HLA matching in 28,376 first unrelated transplants reported to the EBMT Registry with available 6‐locus high‐resolution typing. Mismatches at each locus were quantified and characterised at the allelic, antigenic and functional (antigen‐recognition domain, peptide‐binding motif) levels. 25% of the transplants were performed across one (9/10; *n* = 6053) or more (< 9/10; *n* = 1013) high‐resolution mismatches at the five main HLA loci, a proportion that was markedly higher (43.9%) among transplants performed with post‐transplantation cyclophosphamide (PTCy). Median time from diagnosis to transplant was longer for MMUD compared to 10/10 transplants, but this difference decreased over time (14.9 vs. 11.3 months pre‐2011, *p* = 0.003; 8.1 vs. 7.4 months 2021–2022, *p* = 0.016). Across transplant eras, single class I mismatches were three times more common than class II mismatches. Conversely, matching for HLA‐DPB1 increased from 15% pre‐2011 to 31% in 2021–2022. The landscapes of allelic mismatches differed markedly between HLA loci. For class II, skewed distributions dominated by frequent combinations result in significantly higher frequencies of functional matching compared to class I in both PTCy and non‐PTCy pairs. Our study constitutes the first large‐scale characterisation of real‐world HLA mismatch frequencies in contemporary unrelated HCT, bearing implications for future clinical outcome studies.

## 1 Introduction

Allogeneic haematopoietic cell transplantation (HCT) offers a potentially curative treatment option for many patients with haematological and immunological diseases [1, 2, 3]. Key to the success of this therapeutic approach is sufficient compatibility between the HLA of the recipient and that of the donor. Landmark studies performed at the turn of the century established that each allelic incompatibility at HLA‐A, ‐B, ‐C or ‐DRB1 significantly reduced survival after HCT with unrelated donors [4, 5, 6, 7, 8], mainly because of the increased risk of severe graft‐versus‐host disease (GvHD). Nevertheless, some patients are unable to find a well‐matched donor; for these patients, the risks associated with the use of mismatched related or unrelated donors are weighted against the urgency to transplant, resulting in a proportion of transplants performed across HLA barriers, especially in underserved patient populations [9]. Advances in GvHD prevention and treatment, in particular the advent of post‐transplantation cyclophosphamide (PTCy) [10, 11, 12, 13, 14, 15, 16], have allowed for these transplants to be performed successfully in many patients and the frequency of mismatched transplantation has increased significantly in the last decade and is expected to continue growing [17, 18]. However, contemporary analyses of the prevalence and effects of different types of HLA mismatches are lacking; these are relevant in view of the ongoing changes in clinical practice both in terms of donor selection and HCT protocols and supportive care.

In a recent study [19], we assessed the effects of HLA mismatches on the outcome of contemporary unrelated HCT in a cohort of over 17,000 transplants from the EBMT Registry performed under conventional or PTCy‐based GvHD prophylaxis. Our results showed that HLA incompatibility remains an important factor influencing mortality risks after HCT with unrelated donors, but that this role is mainly driven by mismatches at HLA‐A, ‐B, ‐C and not HLA‐DRB1 or ‐DQB1. It is currently unknown if the nature and the distribution of mismatches at individual HLA loci in contemporary transplantation could explain these differential effects. Here, we set out to investigate this by analysing the frequency of specific mismatch combinations and their functional characteristics in an extended cohort from the EBMT Registry with available 6‐locus, high‐resolution HLA typing for patients and donors. HLA mismatching at each locus was characterised at the allelic, antigenic, antigen‐recognition domain (ARD) and peptide‐binding motif (PBM) levels and the landscapes and functionality of specific mismatches compared across loci.

## 2 Patients and Methods

### 2.1 Study Population and Design

The study included data from adult and paediatric patients who underwent a first HCT from an unrelated bone marrow or peripheral blood stem cell donor reported to the EBMT Registry for which high‐resolution typing at HLA‐A, ‐B, ‐C, ‐DRB1, ‐DQB1 and ‐DPB1 was available for both recipient and donor. Patients included were treated for malignant and non‐malignant diseases using reduced intensity or myeloablative conditioning regimens. GvHD prophylaxis included conventional (i.e., calcineurin inhibitor) and PTCy‐based regimens, as well as the use of anti‐thymocyte globulin (ATG) or alemtuzumab. In our previous study [19], clinical outcomes had been investigated in a subset of this cohort comprised of adult patients treated for haematological malignancies mostly between 2016 and 2020 (*N* = 17,292). Transplant centres reporting pseudonymised pre‐transplant and follow‐up data to the EBMT Registry commit to obtaining informed consent in agreement with the principles of the Declaration of Helsinki and according to the local regulations applicable at the time of data collection. The study was approved by the Cellular Therapy and Immunobiology Working Party of the EBMT.

### 2.2 HLA Typing and Mismatch Classification

HLA typing data provided by the transplant centres and collected in the EBMT database was curated to remove erroneous or outdated nomenclature, and homogenised to second‐field if a higher resolution was available. High‐resolution (i.e., second field) mismatches at HLA‐A, ‐B, ‐C, ‐DRB1, ‐DQB1 and ‐DPB1 were identified and quantified for each patient‐donor pair. If *null* alleles were present, the patient or donor carrying them was considered homozygous for the other allele at the respective locus. For the subsequent analyses, separate calculations were performed for mismatches at the main HLA loci used for donor selection in Europe [20, 21] and those at HLA‐DPB1. HLA mismatches at HLA‐A, ‐B, ‐C, ‐DRB1 and ‐DQB1 were further classified according to their locus and class (i.e., class I vs. class II), as well as their resolution (i.e., second‐field allelic vs. first‐field antigenic) [22]. ARD matching at these loci was assessed according to whether the mismatched alleles belonged to the same P‐group (matched) or not (mismatched). HLA P‐groups are formed by alleles that share the same amino acid sequence in exons 2 and 3 for class I and exon 2 for class II and are hence expected to be functionally equivalent. P‐group lists were downloaded from https://hla.alleles.org/alleles/p_groups.html (IPD‐IMGT/HLA Database version 3.53.0) [23]. Single mismatches at HLA‐B were classified as B‐leader peptide −21 M/T dimorphism‐matched or mismatched as previously described [24]. Single mismatches at HLA‐A, ‐B and ‐C were also classified as PBM‐matched or mismatched in the graft‐versus‐host (GvH) vector as described previously [25]. Similarly, PBM‐GvH matching was extended to single HLA‐DRB1 mismatches by grouping of alleles at this locus into seven PBM groups based on hierarchical clustering of recent immunopeptidomics data [26, 27, 28, 29]. Single and double mismatches at HLA‐DPB1 were classified as permissive or non‐permissive in both 9/10 and 10/10‐matched pairs according to the standard functional distance‐based, bidirectional TCE model [30, 31, 32, 33, 34]. Further classification of permissively mismatched pairs into core and non‐core TCE group 3 mismatches was performed as described previously [35, 36]. Single mismatches at HLA‐DPB1 were also classified as high versus low expression according to the model based on the allele association with the 3′UTR rs9277534 G/A polymorphism as previously reported [37, 38]. TCE‐permissive, high‐expression (TPHE) HLA‐DPB1 mismatches were further distinguished from other single mismatches at this locus based on their combined classification according to the TCE and expression models [39].

### 2.3 Statistical Analysis

HLA allele frequencies in patients and donors were determined by direct counting. Specific mismatched allele combinations were quantified for single‐mismatch pairs and their frequency among mismatched pairs calculated relative to the number of pairs mismatched for that locus. Transplant year was used to classify pairs into different transplant eras (i.e., pre‐2011, 2011–2015, 2016–2020 and 2021–2022). Median time elapsed from initial diagnosis of the underlying disease to transplant was compared between 10/10 and < 10/10 matched pairs stratified by transplant era using a Kruskal–Wallis test followed by Dunn's test for multiple comparisons. *p*‐values were adjusted to account for multiple comparisons. *p*‐values < 0.01 were considered statistically significant. Statistical analyses were performed with Prism (GraphPad Software; version 9.5.1).

## 3 Results

### 3.1 Cohort Characteristics and Overall HLA Mismatch Assessment

The study cohort consisted of 28,376 patients and their respective donors. Most patients were adults (i.e., age ≥ 18 years, 87.7%) treated with peripheral blood haematopoietic cells (83.5%) for acute myeloid or lymphoid leukaemias (54.4%), or myelodysplastic or myeloproliferative neoplasms (22.1%) between 2011 and 2022 (90.8%). Transplants were performed in 329 centres from 34 different countries. About half of the transplants were performed using reduced‐intensity conditioning (49.5%), while the majority used in vivo T‐cell depletion (81.9%). PTCy‐based GvHD prophylaxis was used in 10.2% of the transplants. Additional information on the cohort characteristics is provided in Table 1.

**TABLE 1:** Patient‐, disease‐ and transplant‐related characteristics of the EBMT cohort.

Variable	Groups	*N* = 28,376
Age in years (median, range)	Patients	53.4 (0.1–80)
	Donors	29 (18–77)
	Missing^a^	2001 (7%)
Patient sex	Male	16,747 (59%)
	Female	11,601 (41%)
	Missing	28 (0.1%)
Disease at transplant	AML	9394 (33%)
	MDS	3646 (13%)
	MPN	1689 (6%)
	ALL	3539 (12%)
	Other^b^	10,089 (36%)
	Missing	19 (0.1%)
Graft source	BM	4631 (16%)
	PBSC	23,718 (84%)
	Missing	27 (0.1%)
Conditioning regimen	MAC	13,882 (49%)
	RIC	14,055 (50%)
	Missing	439 (1%)
T‐cell depletion^c^	No	3948 (14%)
	Yes	23,908 (84%)
	Missing	520 (2%)
Conventional GvHD prophylaxis	CsA + MTX	8836 (31%)
	CsA + MMF	5724 (20%)
	CsA	2541 (9%)
	TAC‐based	3423 (12%)
	Other	7194 (26%)
	Missing	353 (1%)
PTCy use	Yes	2890 (10%)
	No	24,837 (88%)
	Missing	649 (2%)
Transplant year	Pre‐2011^d^	2601 (9%)
	2011–2015	4974 (18%)
	2016–2020	13,903 (49%)
	2021–2022	6896 (24%)
	Missing	2 (0%)
Patient‐donor HLA matching	10/10	21,310 (75%)
	9/10	6053 (21%)
	8/10	855 (3.0%)
	< 8/10	158 (0.6%)

Abbreviations: ALL, acute lymphoblastic leukaemia; AML, acute myeloid leukaemia; BM, bone marrow; CsA, cyclosporin A; GvHD, graft‐versus‐host disease; MAC, myeloablative conditioning; MDS, myelodysplastic neoplasm; MMF, mycophenolate mofetil; MPN, myeloproliferative neoplasm; MTX, methotrexate; *N*, number; PBSC, peripheral blood stem cells; PTCy, post‐transplantation cyclophosphamide; RIC, reduced intensity conditioning; TAC, tacrolimus.

^a^ Missing age in 15 patients and 1986 donors.

^b^ Includes Hodgkin and non‐Hodgkin lymphoma (*N* = 2580), other acute leukaemias (*N* = 2502), chronic leukaemias (*N* = 1250), bone marrow failure syndromes (*N* = 1015), MDS/MPN including chronic myelomonocytic leukaemia (*N* = 947), plasma cell disorders (*N* = 762), inherited disorders (*N* = 632), haemoglobinopathies (*N* = 253) and other disorders (*n* = 148).

^c^ In vivo T‐cell depletion by anti‐thymocyte globulin, anti‐T lymphocyte globulin or alemtuzumab.

^d^ Includes 1995–2000, *n* = 62; 2001–2005, *n* = 442; 2006–2010, *n* = 2097.

**FIGURE 1:**
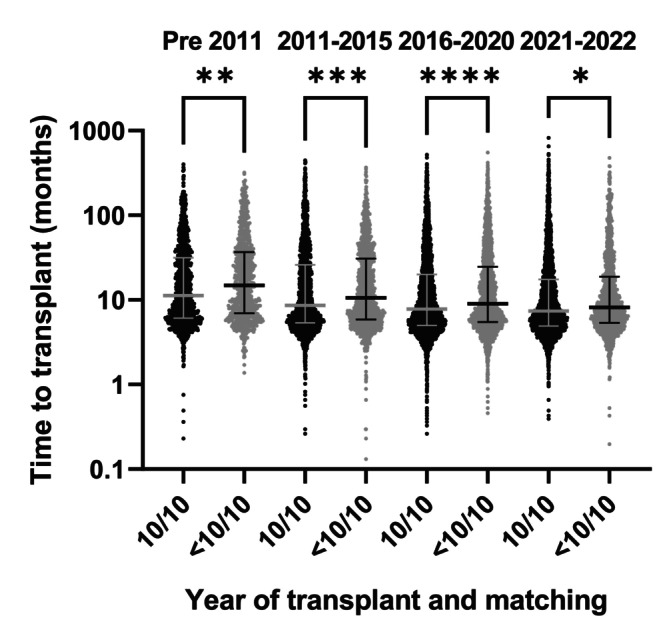
Time from diagnosis to transplant according to HLA matching status. Time from initial diagnosis to transplant in months is plotted for 10/10 (black) versus < 10/10 (grey) pairs in different transplant eras. Bars represent median and interquartile ranges. Adjusted *p* values are derived from a Kruskal–Wallis test with Dunn's multiple comparison test. *p < 0.05, **p < 0.01, ***p < 0.001, ****p < 0.0001.

Overall, 21,310 pairs were 10/10 matched while 7066 (24.9%) transplants were performed across one (9/10; *n* = 6053) or more (< 9/10; *n* = 1013) high‐resolution mismatches at the five main HLA loci. The overall proportion of 10/10 matched transplants (ca. 77%) remained relatively stable from 2016 to 2022 (Table 2). However, mismatched pairs (i.e., < 10/10) were more prevalent among transplants performed with PTCy (43.9%) than in those performed with conventional prophylaxis (22.7%) (Table S1). Median time from diagnosis to transplant was significantly longer for < 10/10‐matched pairs compared to 10/10 transplants, but this difference became smaller across transplant eras (14.9 vs. 11.3 months pre‐2011, *p* = 0.003; 8.1 vs. 7.4 months in 2021–2022, *p* = 0.016; Figure 1). HLA‐DPB1 mismatches were present in 70% and 83.9% of the 10/10 and < 10/10 pairs, respectively. Single HLA‐DPB1 mismatches (46.6% and 51.6% in the 10/10 and 9/10, respectively) were more frequent than double mismatches (23.3% and 31.7% in the 10/10 and 9/10, respectively). Overall, only 6403 (22.6%) pairs were 12/12 matched. Of note, allelic matching for HLA‐DPB1 and the proportion of 12/12 matched transplants became more common in recent years, the former increasing from 15% pre‐2011 to 31% in 2021–2022 (Table 2), as previously noted [40].

**TABLE 2:** HLA matching proportions in the EBMT cohort per transplant era (*N* = 28,376).

Matching	Pre‐2011^a^	2011–2015	2016–2020	2021–2022
10/10	63.9%	72.9%	77.1%	77.0%
12/12	10.4%	15.3%	25.1%	27.3%
HLA‐DPB1 matched^b^	15.4%	19.2%	29.2%	30.9%
HLA‐DPB1 matched (in 10/10)	16.2%	21.1%	32.5%	35.5%
9/10 class I mismatch^c^	18.8%	16.9%	15.0%	15.2%
9/10 class II mismatch^d^	7.3%	6.4%	5.1%	5.4%

^a^ Includes years 1995–2000, *n* = 62; 2001–2005, *n* = 442; 2006–2010, *n* = 2097.

^b^ HLA‐DPB1 allele matching overall (includes 10/10 and < 10/10 matched pairs).

^c^ 9/10 matched with a class I mismatch.

^d^ 9/10 matched with a class II mismatch (i.e., at HLA‐DRB1 or HLA‐DQB1).

### 3.2 HLA Mismatch Frequencies Per Locus, Class and Resolution

**FIGURE 2:**
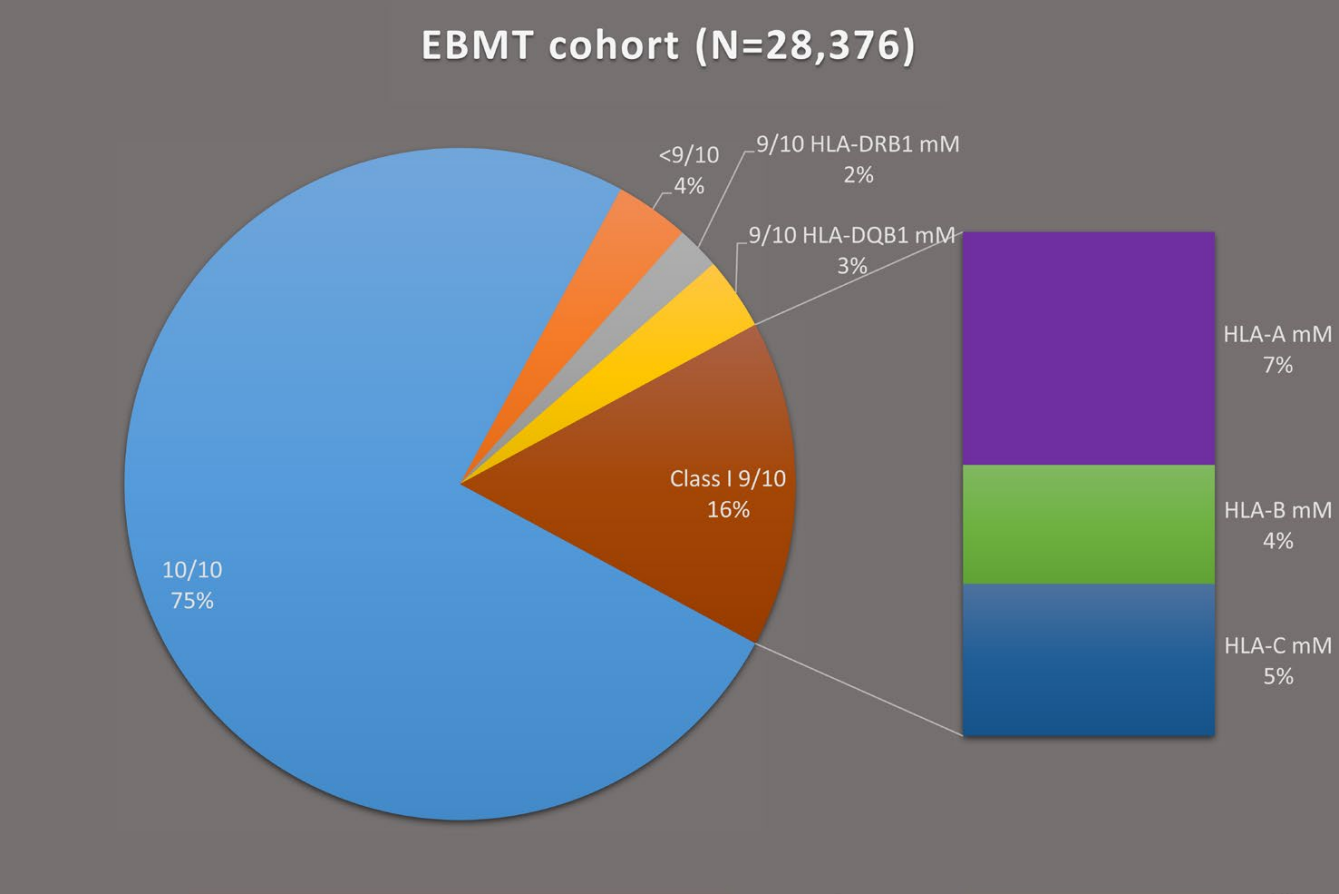
HLA matching status in the EBMT cohort. Shown is the distribution of the number of transplants in the full cohort (*N* = 28,376) according to their allelic matching status at the 5 main HLA loci. mM, mismatch.

Next, we examined in detail the distribution of HLA mismatches among 9/10 and 8/10 matched transplants. Among all 9/10, class I mismatches were three times more common than class II mismatches, a proportion that remained stable across transplant eras (Table 2). Among PTCy pairs, this ratio was even higher at four times (Table S1). Overall, mismatches were most frequent at HLA‐A (*n* = 2063; 34%) and HLA‐C (*n* = 1348; 22.3%), followed by HLA‐B (*n* = 1052; 17.4%) and HLA‐DQB1 (*n* = 996; 16.5%), and rarest at HLA‐DRB1 (*n* = 594; 9.8%) (Figure 2). Single HLA‐A mismatches were more prevalent among PTCy pairs (45.3%) (Table S1). Among the 9/10, the majority of the mismatches (81.0% for HLA‐A, 89.5% for HLA‐B, 80.3% for HLA‐C, 86.0% for HLA‐DRB1 and 77.6% for HLA‐DQB1) were bidirectional, as previously reported [41]. Among the 8/10 matched (*n* = 855), most corresponded to mismatches at two different HLA loci (*n* = 716, 83.7%) as opposed to double mismatches at a single HLA locus (*n* = 139, 16.3%). Only a minority corresponded to mismatches at two class I loci (*n* = 307; 35.9%), while most involved one class I and one class II mismatch (*n* = 422; 49.4%) or two (*n* = 126; 14.7%) class II mismatches, with about half of the latter corresponding to two mismatched alleles at the same locus (Table S2). The prevalence of class II mismatches among the 8/10 pairs, which were not associated with higher mortality risks in the 9/10, could explain the similar overall survival probabilities observed between these subgroups in our previous study [19].

**FIGURE 3:**
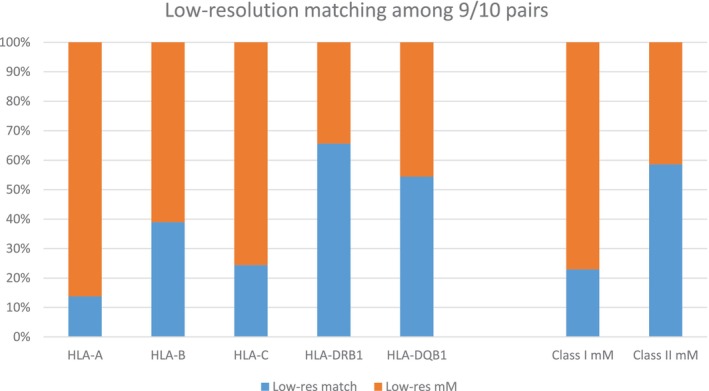
Low‐resolution matching proportions in 9/10 matched pairs. The proportion of low‐resolution (Low‐res, i.e., antigen level) matching among single second‐field allelic mismatched pairs is shown per HLA locus and class. mM, mismatch.

Single HLA class I low‐resolution mismatches (i.e., at the first field or antigen level) were associated with significantly worse survival compared to high‐resolution mismatches in our previous study [19]. Overall, 32.3% of the 9/10 in this extended cohort were matched at low resolution. This proportion was significantly higher for class II, where the majority of allelic mismatches were first‐field matched (58.6% for class II vs. 22.9% for class I; Figure 3). Concordantly, low‐resolution matching rates differed significantly across loci, being highest for HLA‐DRB1 (65.7%) and HLA‐DQB1 (54.4%), followed by HLA‐B (39.0%) and HLA‐C (24.4%), and lowest for HLA‐A (13.7%). Apart from a higher proportion of low‐resolution mismatching among class I‐mismatched pairs, similar proportions were observed for PTCy and non‐PTCy pairs (Table S1).

### 3.3 Landscapes of Allelic Mismatches at Different HLA Loci

**FIGURE 4:**
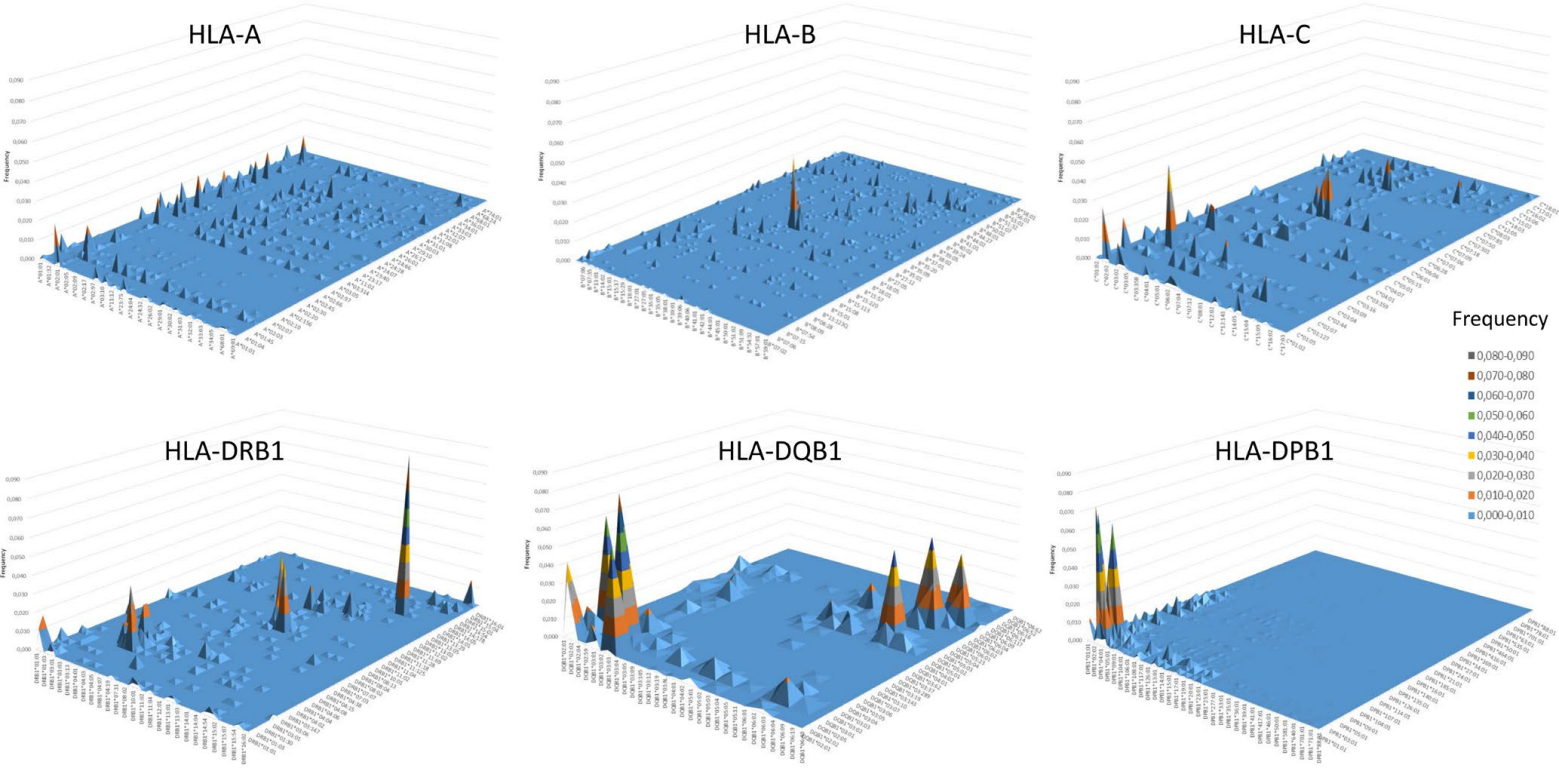
The landscapes of mismatching in unrelated donor HCT differ across HLA loci. The frequency of specific mismatched allele combinations between donor and recipient at each of the main loci is plotted for 9/10 pairs. Single mismatches at HLA‐DPB1 among the 10/10 are also shown. Each peak corresponds to the frequency (*y*‐axis range 0–0.09) of the specific combination of mismatched alleles in the pair, with donor alleles on the left axis and recipient alleles on the right axis. For a higher resolution version of each plot as well as the plot corresponding to single HLA‐DPB1 mismatches among the 9/10, please refer to Figures S1–S7. For a list of all observed single mismatch combinations and their frequencies, please refer to Table S2.

To better understand the distribution of HLA mismatches at the different loci, we recorded the frequencies of mismatched allele combinations for the main loci among the 9/10 matched pairs, and of single mismatches at HLA‐DPB1 in both 10/10 and 9/10 pairs (Table S3; Figure 4; Figures S1–S7). Overall, while mismatches at HLA‐A display a very diverse range of combinations with frequencies up to 2.2%, mismatches at HLA‐DRB1 and HLA‐DQB1 are concentrated in significantly fewer allele pairs reaching frequencies of 7%–9%. For HLA‐DRB1, these are dominated by specific mismatches involving alleles DRB1*14:01‐14:54, DRB1*11:01‐11:04 and DRB1*04:01‐04:04, representing 28.4% of the mismatched pairs. A similar picture is observed for HLA‐DQB1, where mismatches DQB1*03:01‐03:02, DQB1*02:02‐03:03, DQB1*02:01‐02:02, DQB1*05:02‐06:02, DQB1*06:02‐06:03 and DQB1*06:04‐06:09 account for 53.9% of all isolated mismatches at this locus. For HLA‐B and HLA‐C, mismatch diversity lies between the two extremes illustrated above. At HLA‐B, a cluster of combinations around B*35:01, B*35:02, B*35:03 and B*35:08 accounts for 16.6% of all mismatches at this locus. At HLA‐C, mismatches C*03:03‐03:04, C*07:01‐07:02 and C*01:02‐02:02 are the most common combinations, accounting for 17.5% of all mismatches at this locus. Of note, the mismatch allele frequency distributions were similar between PTCy and non‐PTCy pairs (not shown). Specific mismatch frequencies cannot be completely explained by allelic frequencies in patients and donors (Table S4), as common alleles among the subjects are underrepresented among the mismatched alleles in the 9/10. Conversely, rarer alleles in the cohort are overrepresented in the mismatch distributions.

In the case of HLA‐DPB1, both in the 10/10 and 9/10, the distribution of single mismatches is heavily skewed towards combinations involving six very common alleles [42] (i.e., DPB1*01:01, DPB1*02:01, DPB1*03:01, DPB1*04:01, DPB1*04:02 and DPB1*05:01) representing 60.5% and 57.5% of all mismatches at this locus in the 10/10 and 9/10 pairs, respectively (Figure 4 and Figures S6 and S7). Double HLA‐DPB1 mismatches follow a similar pattern, although the large size of the mismatch matrix precludes meaningful analysis (not shown).

### 3.4 Functional HLA Matching Among Allele‐Mismatched Pairs

Next, we examined different algorithms of functional matching in mismatched pairs. First, we evaluated ARD matching, which considers alleles sharing amino acid sequences in this region as having the same peptide‐binding capacities and hence being functionally equivalent. Allelic mismatches that represent ARD matches are overall rare, with only 3.6% of the 9/10 being matched at this level. However, this varied across HLA loci. While only 1% of class I mismatched pairs were ARD matched (0.3% for HLA‐A; 1.1% for HLA‐B; 2.1% for HLA‐C), this was 10.9% of class II mismatched 9/10 (16% for HLA‐DRB1; 7.8% for HLA‐DQB1). ARD matching was similar in PTCy and non‐PTCy pairs (Table S1).

**FIGURE 5:**
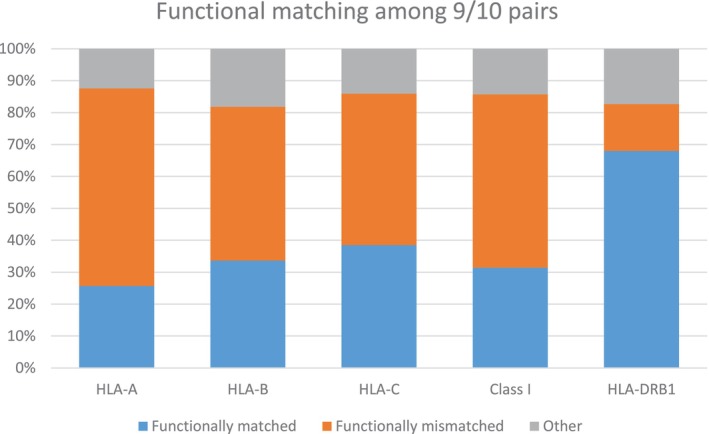
Functional matching among the 9/10 matched differs across HLA loci. The proportions of functionally matched (i.e., allelic mismatches that constitute an ARD and/or a PBM‐GvH match) or mismatched pairs among the 9/10 are shown for each class I and HLA‐DRB1. Other pairs refer to pairs not classifiable with the PBM‐GvH matching algorithm and that are not ARD matches.

**FIGURE 6:**
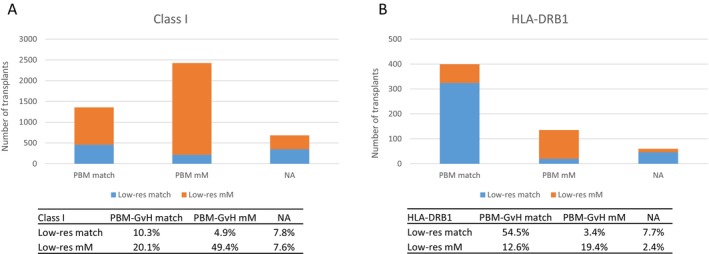
Low‐resolution and PBM‐GvH matching show incomplete overlap in 9/10 patient‐donor pairs. The number and proportion of low‐resolution matched and mismatched pairs is shown for each of the PBM‐GvH matching categories among class I and HLA‐DRB1 single mismatch pairs. mM, mismatch; NA, not applicable (i.e., PBM assignment not possible due to lack of primary immunopeptidomics data for one or more of the allotypes in the mismatch).

We then examined PBM‐GvH matching for class I and HLA‐DRB1. Alleles belonging to the same PBM group share functional characteristics and have overlapping immunopeptidomes [29, 43, 44]. We have shown that class I PBM‐GvH matching confers better outcomes among 9/10 pairs [19, 25]. Of note, while ARD‐matched pairs are considered PBM‐matched, the latter extend beyond HLA P‐groups to all alleles that share similar peptide‐binding patterns. Overall, PBM‐GvH matching assessment was applicable in 85.3% (*N* = 4013) of the 9/10 pairs with allelic mismatches at HLA class I or HLA‐DRB1, and of those 40.7% (*N* = 1755) were PBM‐GvH matched. PBM‐GvH matching proportions differed across classes: while for class I 64% (*n* = 2423) of the classifiable pairs were PBM‐GvH mismatched, this proportion was only 25.3% (*n* = 135) for HLA‐DRB1 single mismatches (Table 3), with similar proportions in both PTCy and non‐PTCy pairs (Table S1). Of note, although frequent (*n* = 116), the mismatch HLA‐C*03:03‐03:04, previously reported as a permissive mismatch [45], represented only 8.4% of the class I PBM‐GvH matched pairs. The higher proportions of ARD and PBM‐GvH matching resulted in significantly higher overall functional matching for HLA‐DRB1 mismatches (68%) compared to class I mismatches (31.4%) among the 9/10 (Figure 5). Finally, the correspondence between low‐resolution matching and PBM‐GvH matching was only partial, and weaker for class I compared to HLA‐DRB1 (Figure 6).

**TABLE 3:** PBM matching^a^ status in the 9/10 per locus (*N* = 5057).^b^

Matching	HLA‐A	HLA‐B	HLA‐C	HLA‐DRB1
PBM‐GvH matched	523 (25.3%)	342 (32.5%)	491 (36.4%)	399 (67.2%)
PBM‐GvH mismatched	1278 (61.9%)	507 (48.2%)	638 (47.3%)	135 (22.7%)
NA^c^	262 (12.7%)	203 (19.3%)	219 (16.2%)	60 (10.1%)

Abbreviations: GvH, graft‐versus‐host; NA, not applicable; PBM, peptide‐binding motif.

^a^ PBM matching in the GvH direction as described in [19, 25, 28].

^b^ Excludes single HLA‐DQB1 mismatched pairs.

^c^ PBM assignment not possible due to lack of primary immunopeptidomics data for one or more of the allotypes in the mismatch.

We also quantified functional matching in the cohort for two additional models, namely HLA‐B leader peptide matching [24], and HLA‐DPB1 TCE matching. When applied to the single HLA‐B mismatched pairs in the cohort (*n* = 1052), only 184 pairs (17.5%) were mismatched for the −21 M/T dimorphism. Of note, the majority (54%) of the patient‐donor‐shared variant distribution corresponded to a TTT combination. The full HLA‐B leader peptide genotype and mismatch combination frequencies can be found in Table S5.

**FIGURE 7:**
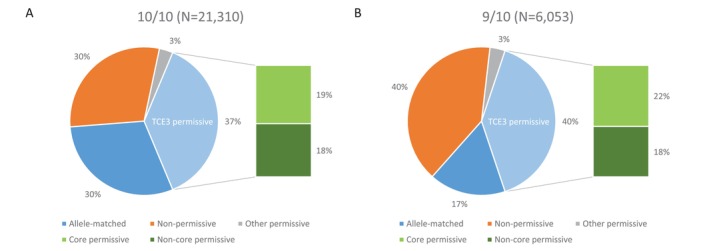
HLA‐DPB1 TCE model matching among well‐matched and 9/10 pairs. The proportions of allelic matches, non‐permissive mismatches and permissive mismatches and their core and non‐core subgroups are shown for the 10/10 and 9/10 pairs. TCE3, T‐cell epitope group 3.

Finally, HLA‐DPB1 mismatches were functionally classified according to the latest developments of the well‐established TCE model [30, 31, 32, 33, 34]. Overall, allele‐matched (26.6%), non‐permissive (32.4%) and permissive (41.1%) pairs represented increasing proportions of the full cohort. Further stratification of the permissive pairs [35, 36] revealed that core (52.8%) and non‐core (47.2%) mismatches represent similar proportions within this subset (Figure 7). The proportions of permissive mismatches were comparable in the 10/10 and < 10/10 pairs (Table 4); conversely, non‐permissive mismatches were more prevalent in the latter (40.3% in the 9/10 vs. 26.6% in the 10/10; Figure 7). GvH non‐core permissive mismatches, which are associated with reduced disease relapse without significant increases in non‐relapse mortality [35], were present in 10.6% of the pairs. An alternative model for the assessment of tolerable mismatches at HLA‐DPB1 based on the level of expression of single GvH mismatches [37, 38] has a partial overlap with the TCE model [46]. This model is applicable in 11,131 (39.2%) pairs in the present cohort, of which 5055 carry a high‐expression mismatch associated with increased GvHD risks (Figure S8). The overlap between subsets of the directional core/non‐core TCE model and TPHE mismatches based on a combination of the TCE and expression models [39] is limited by the applicability of the expression model, as previously observed [40] (Figure S9).

**TABLE 4:** Directional^a^ HLA‐DPB1 TCE matching subsets in the EBMT cohort (*N* = 28,376).

Matching	10/10	9/10	8/10	< 8/10
Allele‐matched	6404 (30.1%)	1010 (16.7%)	109 (12.7%)	21 (13.3%)
Core permissive	4169 (19.6%)	1316 (21.7%)	177 (20.7%)	33 (20.9%)
Non‐core permissive HvG	1581 (7.4%)	433 (7.2%)	68 (8.0%)	7 (4.4%)
Non‐core permissive GvH	2222 (10.4%)	656 (10.8%)	98 (11.5%)	20 (12.7%)
Other permissive	633 (3.0%)	200 (3.3%)	35 (4.1%)	2 (1.3%)
Non‐permissive HvG	2349 (11.0%)	919 (15.2%)	131 (15.3%)	25 (15.8%)
Non‐permissive GvH	3952 (18.5%)	1519 (25.1%)	237 (27.7%)	50 (31.6%)

Abbreviations: GvH, graft‐versus‐host; HvG, host‐versus‐graft.

^a^ Directionality of non‐core permissive and non‐permissive mismatches as described in [35].

## 4 Discussion

In this study, we have performed a comprehensive analysis of HLA mismatch frequencies in a very large cohort of unrelated donor HCT from 34 different countries. Prompted by our previous observation that class I and not class II mismatches associate with worse survival after mismatched HCT [19], we carried out a granular assessment of the different genetic, structural and functional characteristics of individual HLA mismatches at all major loci in a significantly extended cohort. Our results show that (1) one in four transplants included has been performed across main HLA barriers, and that those mismatches are three times more frequent for class I than for class II; (2) that the distribution of specific allelic mismatch combinations differs significantly between HLA loci, with some loci displaying skewed landscapes dominated by a few high‐frequency combinations; and (3) that for class II, this results in significantly higher frequencies of functional (i.e., ARD and PBM) matching compared to class I, potentially contributing to their apparent lack of detrimental effects on survival in our previous study. Of note, these observations did not differ between PTCy and non‐PTCY pairs, despite MMUD being more frequent in the former. In addition, we provide novel observations regarding the frequency of functional models of HLA matching (i.e., B‐leader, class I and HLA‐DRB1 PBM‐GvH, HLA‐DPB1 TCE, expression and TPHE) and their interaction. Considering that our cohort includes mostly (78%) transplants performed in the last decade, our study constitutes a first large‐scale analysis of real‐world HLA mismatching in contemporary HCT.

HLA matching in unrelated HCT remains a main recommendation for the success of this therapy [1, 2, 8, 47] based on evidence from numerous independent retrospective studies [6, 24, 48, 49]. However, this notion is currently being challenged by the promising results using mismatched related and unrelated donors, in particular with PTCy‐based GvHD prophylaxis [15, 16, 50]. Hence, the use of MMUD, already frequent in the pre‐PTCy era as reflected by our data, is rapidly increasing [18], as it is considered a safe option that reduces the inequalities in access to transplant due to donor availability [16, 51, 52]. Our previous study confirmed a role for HLA mismatches, in particular those in class I, in survival after unrelated HCT with and without PTCy [19], although the role of HLA in the latter setting is still controversial [16, 53]. The results presented here in terms of specific mismatch combinations prevalent among class I versus class II mismatched pairs could potentially underlie the differences seen in clinical outcomes between HLA loci. Whether these observations will be affected by the increasing proportion of transplants performed under PTCy [18] will have to be assessed in the near future, once sufficient numbers of transplants are accumulated. In particular, this will allow us to investigate more thoroughly the intriguing hypothesis that mismatches with higher immunogenicity, considered deleterious or non‐permissive under conventional GvHD prophylaxis, could actually be beneficial due to better disease control under PTCy [28, 54, 55]. Until then, our observations in this context are to be considered hypothesis‐generating and recommendations on donor selection for HCT under PTCy based on functional matching should be taken with caution.

The mismatch frequencies observed in our study are likely the result of the combined effects of the underlying allelic frequencies at each locus and the linkage disequilibrium between alleles at different loci in the population from which the patients are drawn, as well as clinical practice in the process of donor selection. Alleles that are more frequent in the population are more likely to be matched, and hence tend to be underrepresented in the mismatch distributions. On the other hand, when two alleles are in strong linkage with the same frequent allele at another locus, mismatches involving the two linked alleles will be enriched in the mismatch distribution for that locus. When a mismatch is unavoidable, active prioritisation of specific mismatches by the transplant teams could further increase the frequencies of specific combinations considered immunologically equivalent [56] or that belong to the same family and are considered to be of low immunogenicity [57]. It is tempting to think that the reason why HLA‐DRB1 mismatches were associated with worse survival compared to 10/10 pairs in some studies [4, 6, 58], but not in others [24, 57] including ours [19] might reflect different mismatch combinations at this locus present in the different cohorts. Similarly, differing prevalence of well‐tolerated mismatches at HLA‐C (e.g., C*03:03‐03:04) might explain apparently contradicting results in previous studies [45, 59] regarding allelic versus antigenic mismatches at this and other loci [6, 19, 60]. Analysis of the distribution of specific mismatched alleles as presented here could facilitate the comparison and interpretation of the results of different clinical studies examining the role of HLA mismatches in unrelated HCT.

Immunopeptidome divergence between mismatched HLA molecules has been described as a main factor modulating alloreactivity [61] and validated by us as a biomarker for risk stratification and intelligent donor selection in unrelated HCT [19, 25, 35, 36, 62]. Together, ARD and PBM‐GvH matching constitute a functional level of matching based on this principle. Our results show that, as a result of the mismatch distributions discussed above, this functional matching is significantly higher among HLA‐DRB1 mismatches compared to class I mismatches, potentially explaining their lack of clinical association with HCT outcome. Of note, the observation that HLA class I allelic mismatches are less deleterious than antigenic low‐resolution mismatches in our previous study [19] might be explained by its partial correlation with PBM‐GvH matching, as demonstrated here. Future studies should consider using functional matching as the preferred method for the assessment of the role of HLA mismatch in allogeneic HCT, as previously suggested [57].

Despite constituting to our knowledge the largest and most comprehensive analysis of real‐world HLA mismatch frequencies in contemporary unrelated HCT, our study is limited by the constraints of retrospective transplant registry data. This includes the fact that we focused on pairs with full typing data available, excluding transplants with missing data at any HLA locus. This could potentially result in bias towards certain transplant centres or patient populations. In addition, we did not analyse HLA data at higher resolutions (i.e., third and fourth fields), which has been associated with HCT outcome [63] and might provide further insights into HLA mismatch distributions among formally matched pairs. Moreover, we cannot exclude the possibility that some of the HLA data reported by the contributing transplant centres might have been reduced to P‐groups at submission. It should also be noted that our data are only partially informative for HLA population genetics, since allele frequencies might be biased by matching and be skewed towards HLA types from patients with hematologic malignancies. Moreover, the fact that information on ethnicity is unavailable for donors and the majority (84%) of patients in this cohort, precludes an analysis of its role in the findings presented. Considering that MMUD transplantation is rapidly expanding precisely in populations of non‐European origin [50, 64, 65], future studies should address potential differences in HLA mismatch patterns across different continental ancestry groups [66]. Finally, testing of the clinical effects of functional matching for class II observed here is limited by the low numbers of transplants after selection on other clinical variables. These studies are warranted in order to further elucidate the implications of functional HLA matching, especially with the advent of PTCy as GvHD prophylaxis.

In conclusion, our study provides a relevant characterisation of real‐world HLA mismatch frequencies in contemporary unrelated HCT, highlighting the opportunities to favour frequent, better‐tolerated (i.e., functionally matched) mismatches during donor selection to ensure the best possible outcome for all patients. Specific HLA mismatch frequency and functional matching should be monitored prospectively in the PTCy era, in order to improve our understanding of HLA immunobiology in current clinical practice.

## Author Contributions

E.A.‐B. and K.F. designed the study. T.G.‐D., R.P.L., U.S., N.K., I.Y.‐A., M.Z., G.C., C.C., E.T., V.D., J.P., L.W., S.G.E.M. and M.T. contributed clinical data. E.F.B., P.C. and E.A.‐B. performed statistical analyses. E.A.‐B., K.F., A.R. and J.D.H. analysed and interpreted data. E.A.‐B. drafted the manuscript. All authors participated in manuscript writing and review, and provided final approval of the manuscript.

## Funding

This work was supported by Deutsche Knochenmarkspenderdatei (DKMS) (DKMS‐SLS‐JHRG‐2021‐02); José Carreras Leukämie‐Stiftung (DJCLS 17R/2023); Dr. Werner Jackstädt‐Stiftung; Joseph Senker Stiftung.

## Ethics Statement

Transplant centres reporting pseudonymised pre‐transplant and follow‐up data to the EBMT Registry commit to obtaining informed consent in agreement with the principles of the Declaration of Helsinki and according to the local regulations applicable at the time of data collection. The study was approved by the Cellular Therapy and Immunobiology Working Party of the EBMT.

## Conflicts of Interest

E.T. declares honoraria/speaker fees from Autolus, Astellas, BMS/Cellgen, Janssen, Jazz, Kite/Gilead, SOBI, Pfizer, Vertex, none are related to this work. F.M. reports lecture honoraria from Therakos/Mallinckrodt, BMS, MSD, Sanofi, Novartis, Astra Zeneca and JAZZ Pharmaceuticals, all outside the submitted work. The other authors declare no conflicts of interest.

## Supporting information


**Data S1:** tan70637‐sup‐0001‐Supinfo.docx.

**Data S2:** tan70637‐sup‐0002‐Tables‐3‐and‐4.xlsx.

## Data Availability

Data are available on request from the EBMT Cellular Therapy and Immunobiology Working Party Chair (ctiwp@ebmt.org).
