# Supplementary material for: Landscapes of HLA Mismatching in Contemporary Unrelated Haematopoietic Cell Transplantation
Source: HLA. 2026 Mar 11;107(3):e70637. doi: 10.1111/tan.70637 (PMC12978212; doi:10.1111/tan.70637)

**Landscapes of HLA mismatching in contemporary unrelated hematopoietic cell transplantation**

Short Title: HLA mismatch landscapes in unrelated HCT

Esteban Arrieta-Bolaños^1,2^, Edouard F. Bonneville^3,4^, Pietro Crivello^1^, Tobias Gedde-Dahl^5^, Régis Peffault de Latour^6^, Urpu Salmenniemi^7^, Nicolaus Kröger^8^, Ibrahim Yakoub-Agha^9^, Marco Zecca^10^, Goda Choi^11^, Charles Crawley^12^, Eleni Tholouli^13^, Valérie Dubois^14^, Juha Peräsaari^15^, Lotte Wieten^16^, Steven G. E. Marsh^17^, Mats Bengtsson^18^, Jorinde D. Hoogenboom^4^, Jürgen Kuball^19#^, Florent Malard^20#^, Annalisa Ruggeri^21*#^, and Katharina Fleischhauer^1,2*^

^1^Institute for Experimental Cellular Therapy, University Hospital Essen, Essen, Germany

^2^German Cancer Consortium (DKTK), partner site Essen/Düsseldorf, Essen, Germany

^3^Department of Biomedical Data Sciences, LUMC, Leiden, The Netherlands

^4^EBMT Leiden Study Unit, Leiden, The Netherlands

^5^Oslo University Hospital, Rikshospitalet, Oslo, Norway

^6^Saint-Louis Hospital, BMT Unit, Paris, France

^7^HUCH Comprehensive Cancer Center, Helsinki, Finland

^8^University Medical Center Hamburg, Hamburg, Germany

^9^CHU de Lille, Univ Lille, INSERM U1286, 59000 Lille, France

^10^San Matteo Pavia Transplant Programme, Fondazione IRCCS Policlinico San Matteo, Pavia, Italy

^11^University Medical Center Groningen, University of Groningen, Groningen, The Netherlands

^12^Addenbrookes Hospital Cambridge, Cambridge, United Kingdom

^13^Manchester Royal Infirmary, Manchester, United Kingdom

^14^Histocompatibility Laboratory, EFS Lyon, Lyon, France

^15^Clinical Laboratory Services, Histocompatibility Testing, Finnish Red Cross Blood Service, Vantaa, Finland

^16^Transplantation Immunology, Maastricht University Medical Center, Maastricht, The Netherlands

^17^UCL Cancer Institute, London, United Kingdom

^18^Department of Immunology, Genetics and Pathology, Uppsala University, Uppsala, Sweden

^19^Department of Hematology, University Medical Center Utrecht, Utrecht, The Netherlands

^20^Sorbonne Université, Centre de Recherche Saint-Antoine (CRSA) INSERM UMRs938, Service d'Hématologie Clinique et Thérapie Cellulaire, Hôpital Saint-Antoine, AP-HP Paris France

^21^San Raffaele Scientific Institute, Hematology and Bone Marrow Transplantation Unit, Milan, Italy

*Shared senior authorship

#On behalf of the CTIWP of the EBMT

Corresponding author:

Esteban Arrieta-Bolaños, PhD

Institute for Experimental Cellular Therapy, University Hospital Essen, Germany

Email: esteban.arrieta-bolanos@uk-essen.de

Telephone: +49 201 723 4584

Fax: +49 201 723 4546

**This file includes:**

Supplemental Tables: 1-5

Supplemental Figures: 1-9

**SUPPLEMENTAL TABLES**

| **Table S1. HLA matching in the full cohort and in the subsets with and without PTCy.** | | | | | | |
| --- | --- | --- | --- | --- | --- | --- |
|  | **All** | | **PTCy** | | **No-PTCy** | |
| Matching status | N | % | N | % | N | % |
| 12/12 | 6403 | 22.6 | 492 | 17.0 | 5911 | 23.2 |
| 10/10 | 21310 | 75.1 | 1622 | 56.1 | 19688 | 77.3 |
| 9/10 | 6053 | 21.3 | 1089 | 37.7 | 4964 | 19.5 |
| <9/10 | 1013 | 3.6 | 179 | 6.2 | 834 | 3.3 |
| single class I MM | 4463 | 73.7 | 878 | 80.6 | 3585 | 72.2 |
| single class II MM | 1590 | 26.3 | 211 | 19.4 | 1379 | 27.8 |
|  |  |  |  |  |  |  |
| **Among 9/10 pairs** | | | | | | |
| Mismatched locus |  | | | | | |
| HLA-A | 2063 | 34.1 | 493 | 45.3 | 1570 | 31.6 |
| HLA-B | 1052 | 17.4 | 198 | 18.2 | 854 | 17.2 |
| HLA-C | 1348 | 22.3 | 187 | 17.2 | 1161 | 23.4 |
| HLA-DRB1 | 594 | 9.8 | 93 | 8.5 | 501 | 10.1 |
| HLA-DQB1 | 996 | 16.5 | 118 | 10.8 | 878 | 17.7 |
|  |  |  |  |  |  |  |
| Class I MM, low-res matched | 1022 | 22.9 | 135 | 15.4 | 887 | 24.7 |
| Class I MM, low-res mismatched | 3441 | 77.1 | 743 | 84.6 | 2698 | 75.3 |
| Class II MM, low-res matched | 932 | 58.6 | 122 | 57.8 | 810 | 58.7 |
| Class II MM, low-res mismatched | 658 | 41.4 | 89 | 42.2 | 569 | 41.3 |
|  |  |  |  |  |  |  |
| Class I MM, ARD matched | 47 | 1.1 | 5 | 0.6 | 42 | 1.2 |
| Class I MM, ARD mismatched | 4416 | 98.9 | 873 | 99.4 | 3543 | 98.8 |
| Class II MM, ARD matched | 175 | 11.0 | 18 | 8.5 | 157 | 11.4 |
| Class II MM, ARD mismatched | 1415 | 89.0 | 193 | 91.5 | 1222 | 88.6 |
|  |  |  |  |  |  |  |
| Class I MM, PBM matched | 1356 | 30.4 | 247 | 28.1 | 1109 | 30.9 |
| Class I MM, PBM mismatched | 2423 | 54.3 | 500 | 56.9 | 1923 | 53.6 |
| Class I MM, NA | 684 | 15.3 | 131 | 14.9 | 553 | 15.4 |
| HLA-DRB1 MM, PBM matched | 399 | 67.2 | 60 | 64.5 | 339 | 67.7 |
| HLA-DRB1 MM, PBM mismatched | 135 | 22.7 | 25 | 26.9 | 110 | 22.0 |
| HLA-DRB1 MM, NA | 60 | 10.1 | 8 | 8.6 | 52 | 10.4 |
|  |  |  |  |  |  |  |
| **Total** | **28,376** | **100** | **2,890** | **100** | **25,486** | **100** |
| ARD, antigen-recognition domain; low-res, low-resolution (i.e. first field); MM, mismatch; NA, not-applicable; PBM, peptide-binding motif; PTCy, post-transplantation cyclophosphamide. | | | | | | |

| **Table S2. Distribution of double mismatches among 8/10 pairs in the cohort.** | | | | | | | | | | | | | | | | |
| --- | --- | --- | --- | --- | --- | --- | --- | --- | --- | --- | --- | --- | --- | --- | --- | --- |
| **8/10** | **Mismatched HLA loci** | | | | | | | | | | | | | | |  |
| **Class** | A | AB | AC | A  DQ | A  DR | B | BC | B  DQ | B  DR | C | C  DQ | C  DR | DQ | DR | DR  DQ | **Total** |
| **Class I** | 34 | 68 | 60 |  |  | 13 | 97 |  |  | 35 |  |  |  |  |  | **307** |
| **Class I + Class II** |  |  |  | 126 | 63 |  |  | 81 | 37 |  | 92 | 23 |  |  |  | **422** |
| **Class II** |  |  |  |  |  |  |  |  |  |  |  |  | 40 | 17 | 69 | **126** |
| **Total** | **34** | **68** | **60** | **126** | **63** | **13** | **97** | **81** | **37** | **35** | **92** | **23** | **40** | **17** | **69** | **855** |

**Table S3a. Frequency of HLA mismatch combinations among 9/10 matched pairs. (see Excel file)**

**Table S3b. Frequency of HLA-DPB1 single mismatch combinations among 10/10 and 9/10 matched pairs. (see Excel file)**

**Table S4a. HLA allele frequencies in patients and donors in the EBMT cohort (2N=56,752). (see Excel file)**

**Table S4b. HLA allele frequencies in patients and donors in the EBMT cohort (2N=56,752). (see Excel file)**

| **Table S5. HLA-B leader peptide genotype and patient-donor-shared combinations in the 9/10 HLA-B mismatched pairs (n=1,052)** | | |
| --- | --- | --- |
| **Genotype** | **Patients (%)** | **Donors (%)** |
| MM | 53 (5.0) | 55 (5.2) |
| MT | 342 (32.5) | 364 (34.6) |
| TT | 645 (61.3) | 630 (59.9) |
| NA^1^ | 12 (1.1) | 3 (0.3) |
|  |  |  |
| **Pair** | **N** | **%** |
| TTT | 568 | 54.0 |
| TTM | 188 | 17.9 |
| TMT | 76 | 7.2 |
| MMT | 69 | 6.6 |
| MTT | 59 | 5.6 |
| MMM | 29 | 2.8 |
| TMM | 25 | 2.4 |
| MTM | 24 | 2.3 |
| NA^1^ | 14 | 1.3 |
| **Total** | **1052** | **100** |
| ^1^NA, not applicable since the alleles lacked exon 1 sequence on the IPD-IMGT/HLA database | | |

**SUPPLEMENTAL FIGURE LEGENDS**

**Fig S1. The landscape of single mismatches at HLA-A in the EBMT cohort.** The frequency of specific mismatched allele combinations between donor and recipient are plotted for 9/10 pairs with a single HLA-A mismatch (N=2,063). Each peak corresponds to the frequency (y-axis range 0-0.09) of the specific combination of mismatched alleles in the pair, with donor alleles on the left axis and recipient alleles on the right axis. Some allele labels are omitted for clarity. This figure represents an expanded version of the plot included in Figure 4.

**Fig S2. The landscape of single mismatches at HLA-B in the EBMT cohort.** The frequency of specific mismatched allele combinations between donor and recipient are plotted for 9/10 pairs with a single HLA-B mismatch (N=1,052). Each peak corresponds to the frequency (y-axis range 0-0.09) of the specific combination of mismatched alleles in the pair, with donor alleles on the left axis and recipient alleles on the right axis. Some allele labels are omitted for clarity. This figure represents an expanded version of the plot included in Figure 4.

**Fig S3. The landscape of single mismatches at HLA-C in the EBMT cohort.** The frequency of specific mismatched allele combinations between donor and recipient are plotted for 9/10 pairs with a single HLA-C mismatch (N=1,348). Each peak corresponds to the frequency (y-axis range 0-0.09) of the specific combination of mismatched alleles in the pair, with donor alleles on the left axis and recipient alleles on the right axis. Some allele labels are omitted for clarity. This figure represents an expanded version of the plot included in Figure 4.

**Fig S4. The landscape of single mismatches at HLA-DRB1 in the EBMT cohort.** The frequency of specific mismatched allele combinations between donor and recipient are plotted for 9/10 pairs with a single HLA-DRB1 mismatch (N=594). Each peak corresponds to the frequency (y-axis range 0-0.09) of the specific combination of mismatched alleles in the pair, with donor alleles on the left axis and recipient alleles on the right axis. Some allele labels are omitted for clarity. This figure represents an expanded version of the plot included in Figure 4.

**Fig S5. The landscape of single mismatches at HLA-DQB1 in the EBMT cohort.** The frequency of specific mismatched allele combinations between donor and recipient are plotted for 9/10 pairs with a single HLA-DQB1 mismatch (N=996). Each peak corresponds to the frequency (y-axis range 0-0.09) of the specific combination of mismatched alleles in the pair, with donor alleles on the left axis and recipient alleles on the right axis. Some allele labels are omitted for clarity. This figure represents an expanded version of the plot included in Figure 4.

**Fig S6. The landscape of single mismatches at HLA-DPB1 among 10/10 pairs in the EBMT cohort.** The frequency of specific mismatched allele combinations between donor and recipient are plotted for 10/10 pairs with a single HLA-DPB1 mismatch (N=9,940). Each peak corresponds to the frequency (y-axis range 0-0.09) of the specific combination of mismatched alleles in the pair, with donor alleles on the left axis and recipient alleles on the right axis. Some allele labels are omitted for clarity. This figure represents an expanded version of the plot included in Figure 4.

**Fig S7. The landscape of single mismatches at HLA-DPB1 among 9/10 pairs in the EBMT cohort.** The frequency of specific mismatched allele combinations between donor and recipient are plotted for 9/10 pairs with an additional single HLA-DPB1 mismatch (N=3,122). Each peak corresponds to the frequency (y-axis range 0-0.09) of the specific combination of mismatched alleles in the pair, with donor alleles on the left axis and recipient alleles on the right axis. Some allele labels are omitted for clarity.

**Fig S8. Classification of HLA-DPB1 mismatches in the EBMT cohort according to the expression model.** The proportion of single GvH HLA-DPB1 mismatches with high (G) and low (A) expression as determined by the linkage between the mismatched allele and the 3’UTR rs9277534 G/A polymorphism is shown. The expression model is not applicable in pairs with double mismatches, single mismatches in the host-versus-graft (HvG) direction and in a few pairs with mismatches alleles lacking a known linkage to the rs9277534 G/A polymorphism (not reported).

**Fig S9. Overlap between TPHE and core/non-core subsets among permissive HLA-DPB1 mismatches in the EBMT cohort.** Permissive mismatches at HLA-DPB1 (N=11,650) in the full cohort were classified as TCE-permissive and high-expression (TPHE) and as directional core/non-core subsets as previously described. Shown is the overlap of each category (TPHE, upper half; directional core/non-core TCE model, lower half) among permissively mismatched pairs. 41% of the pairs cannot be classified for TPHE mismatches since the expression model is not applicable (NA). TPLE, single TCE-permissive, low expression mismatch; Other, other permissive pairs not involving exclusively TCE group 3 alleles.


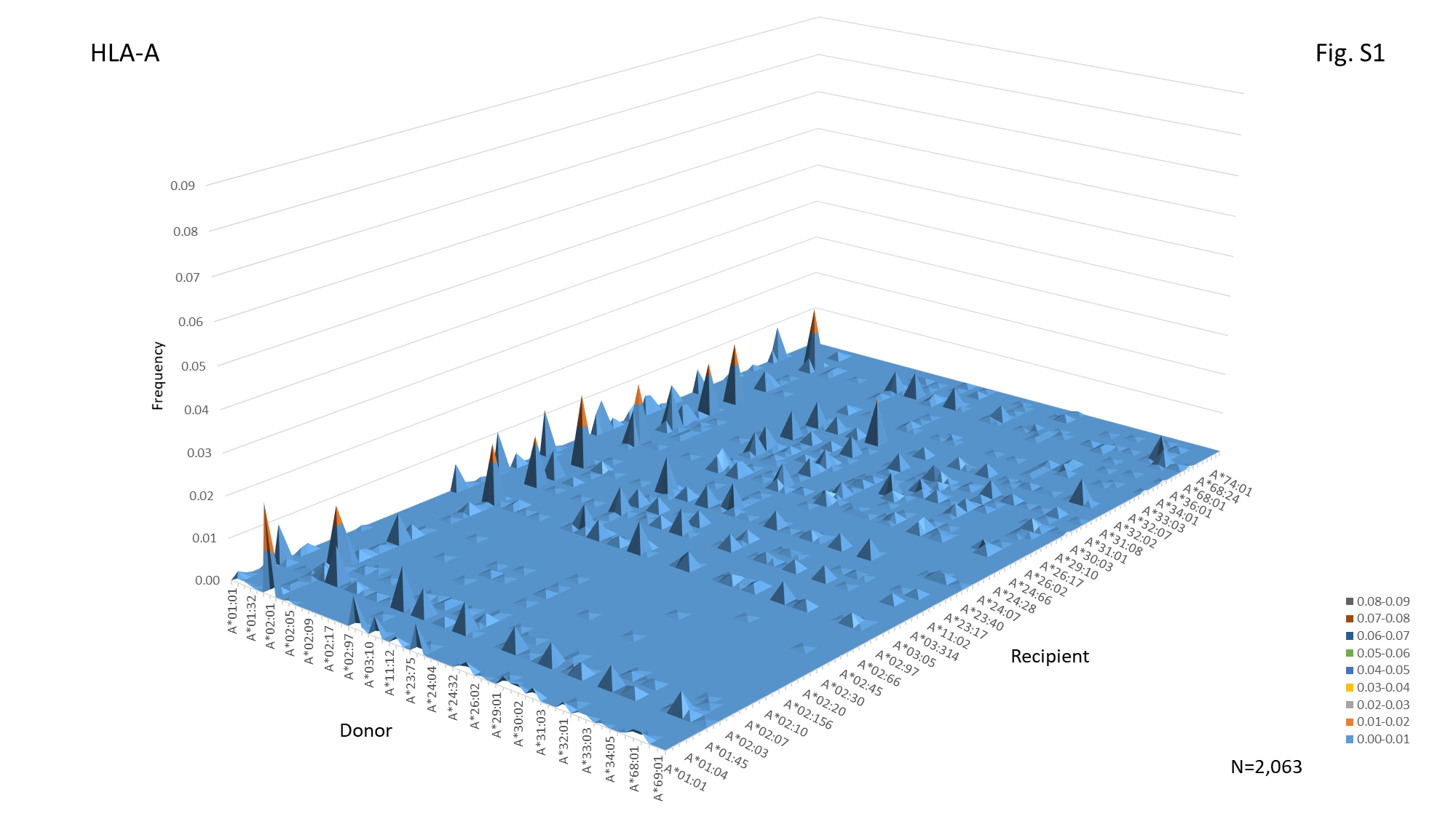


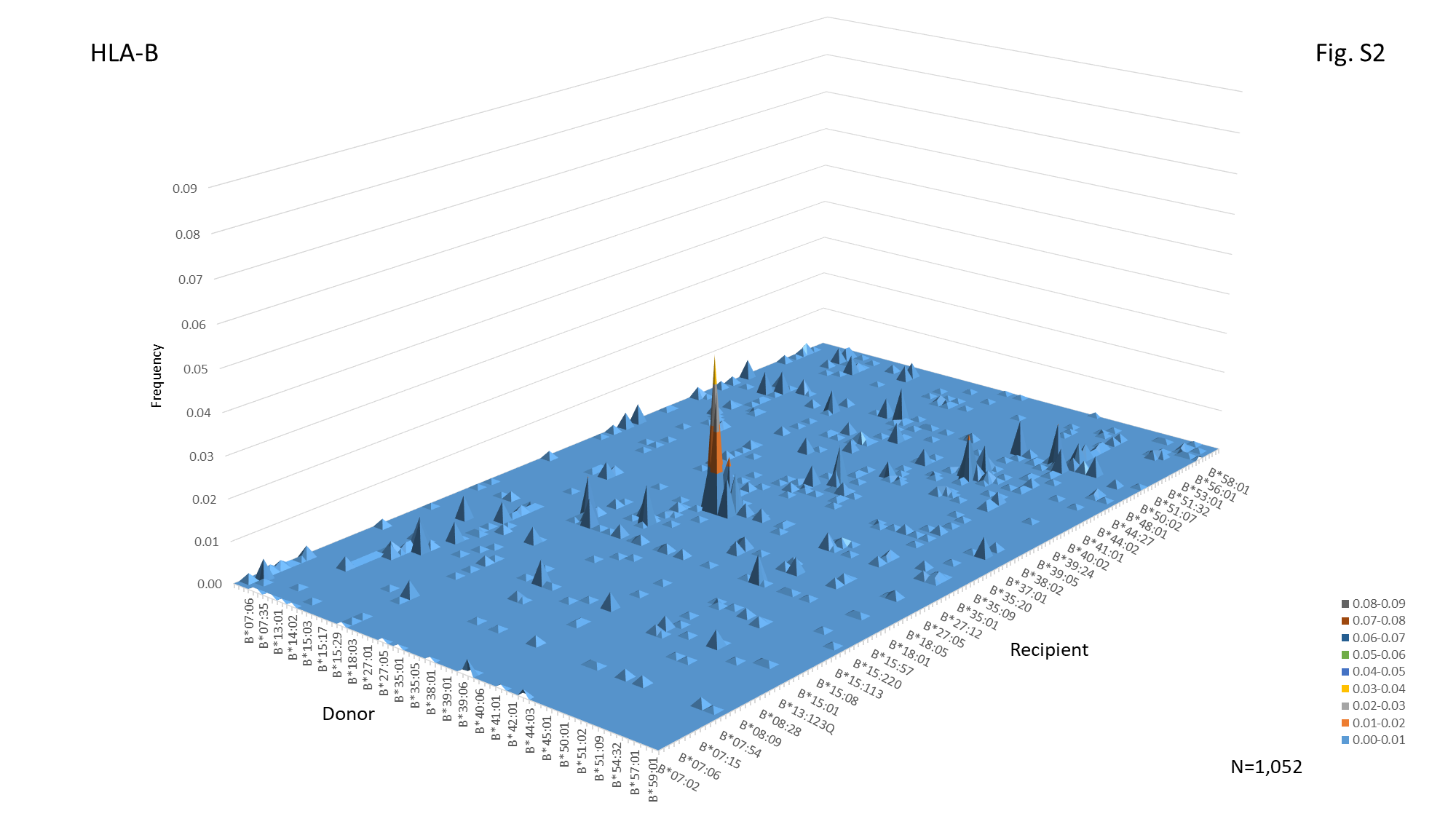


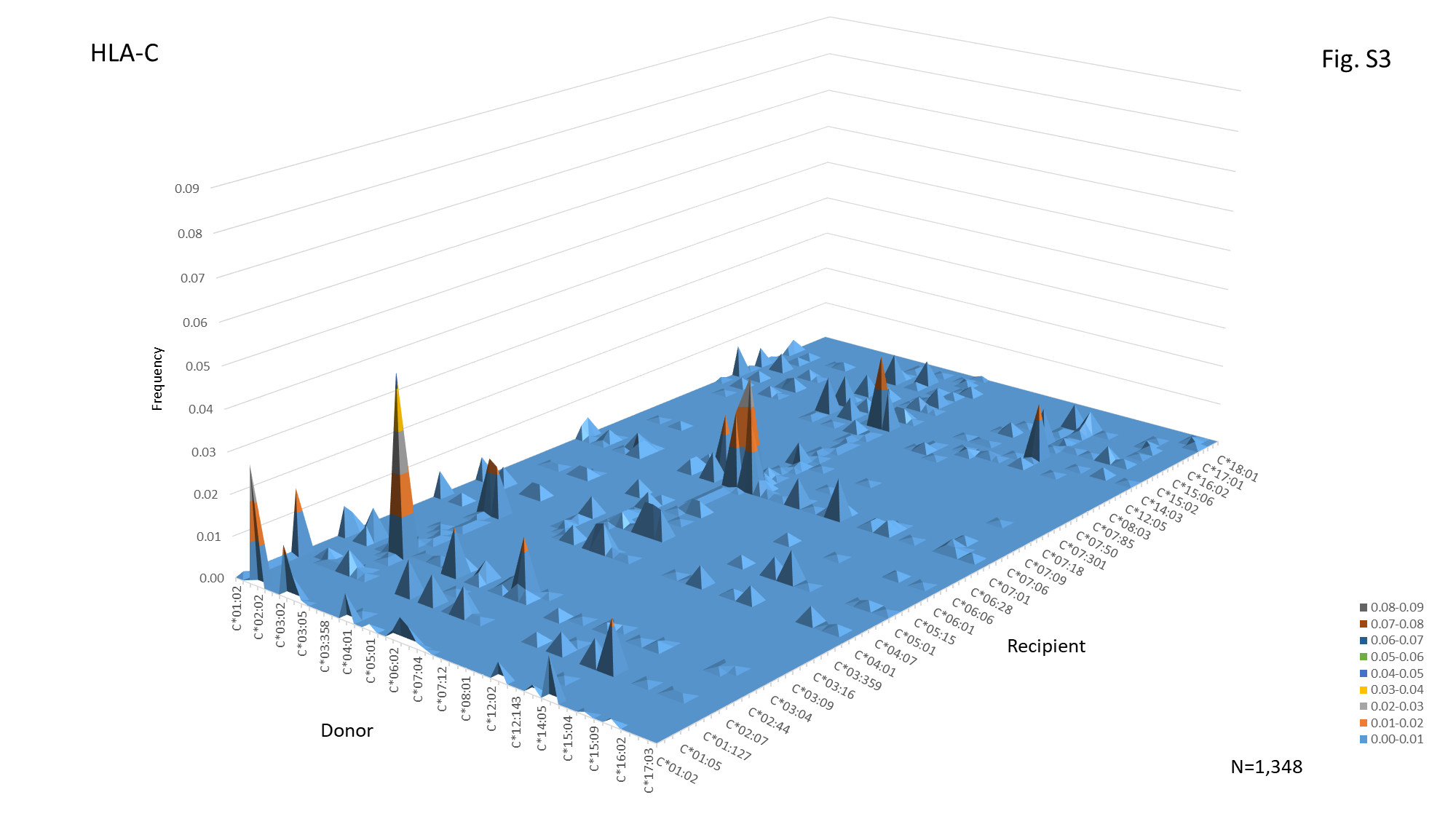


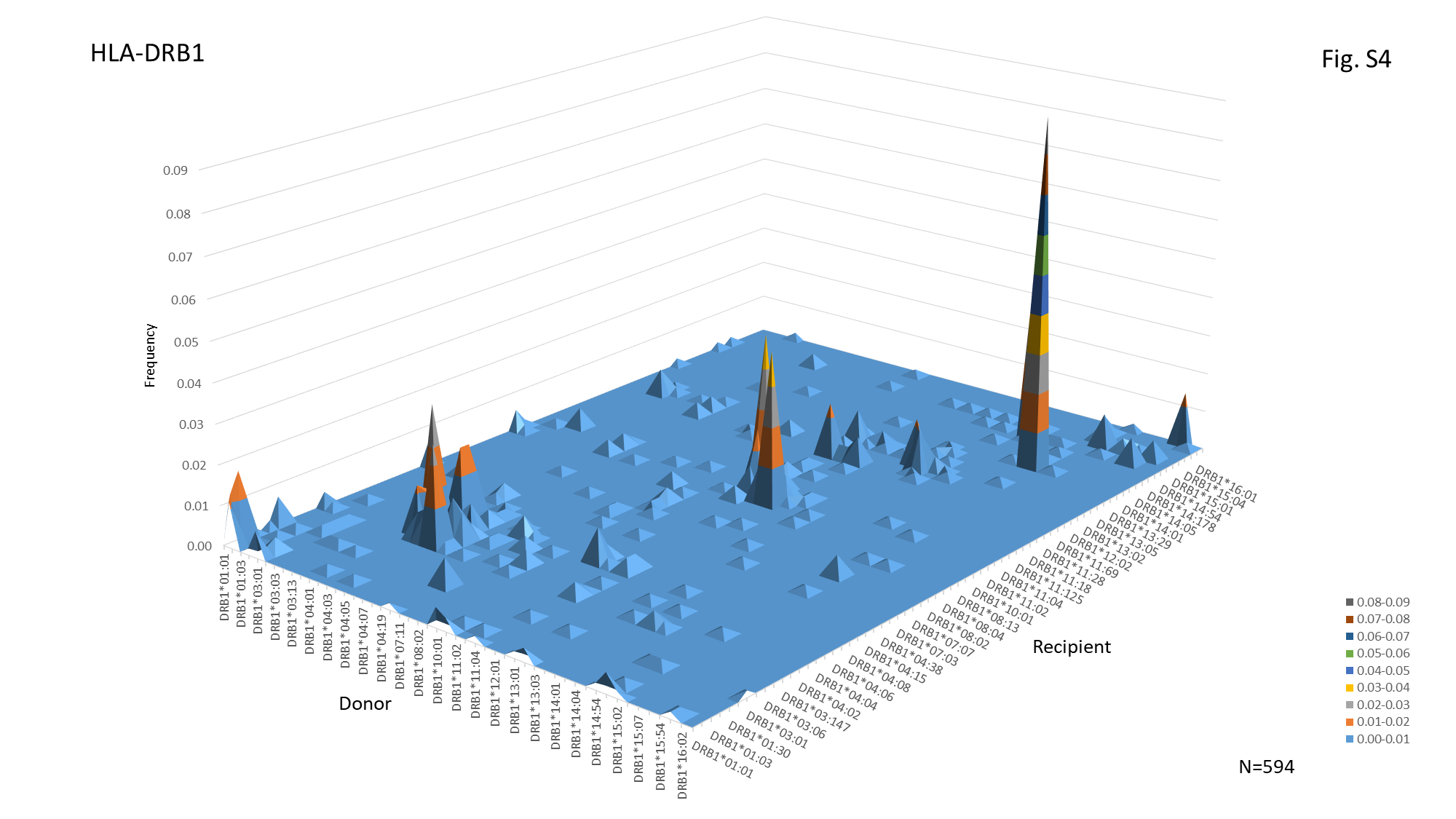


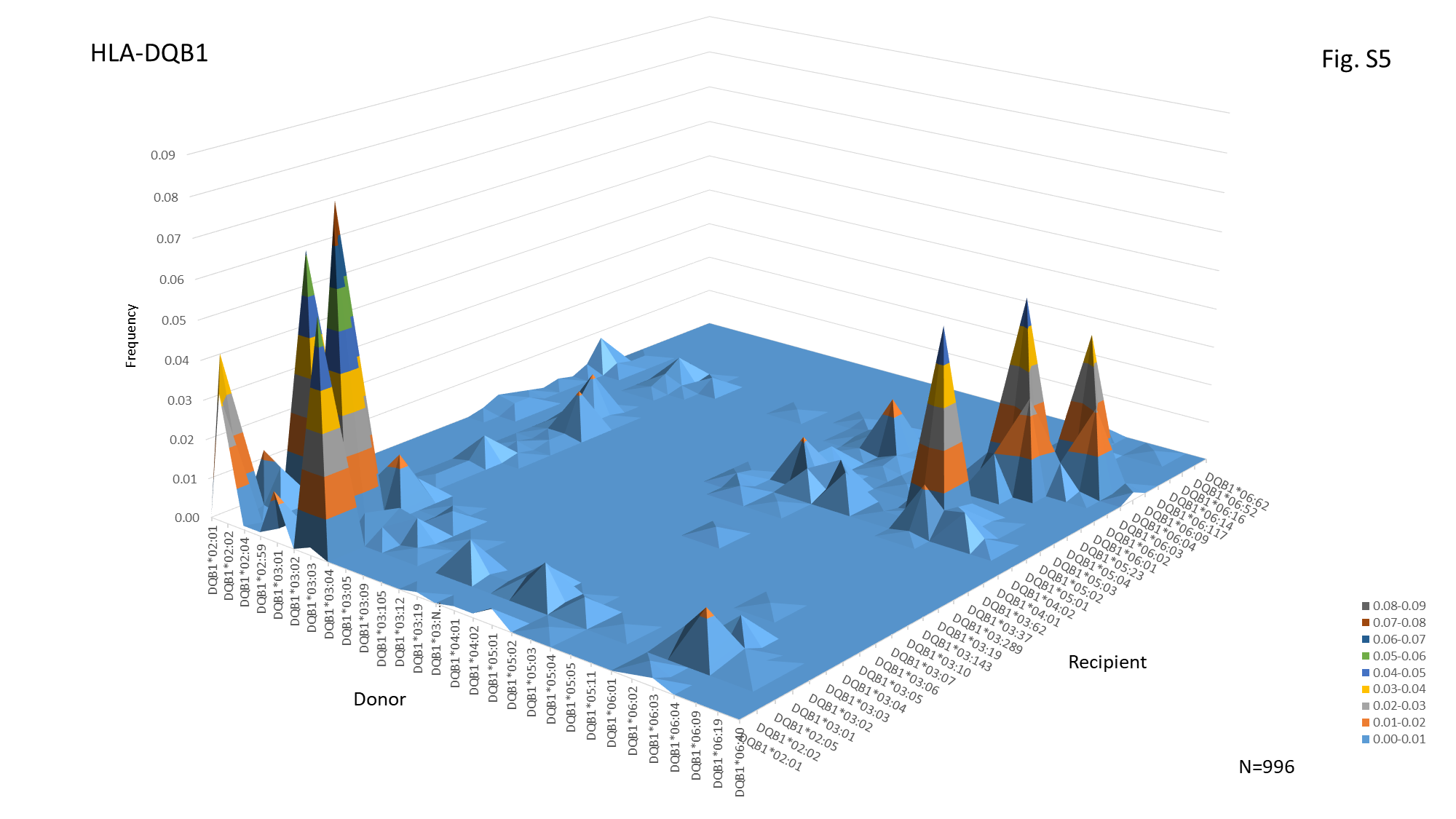


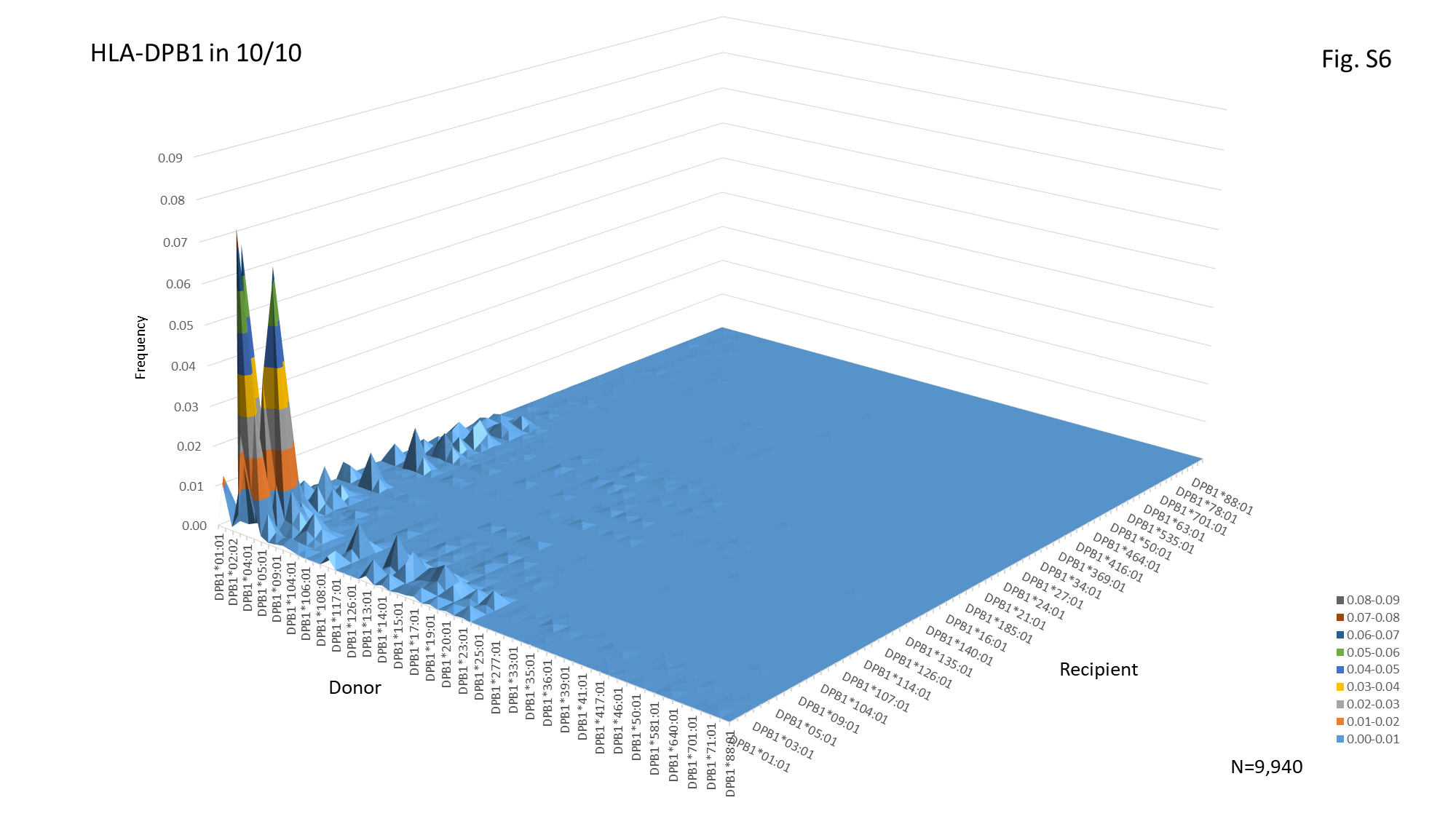


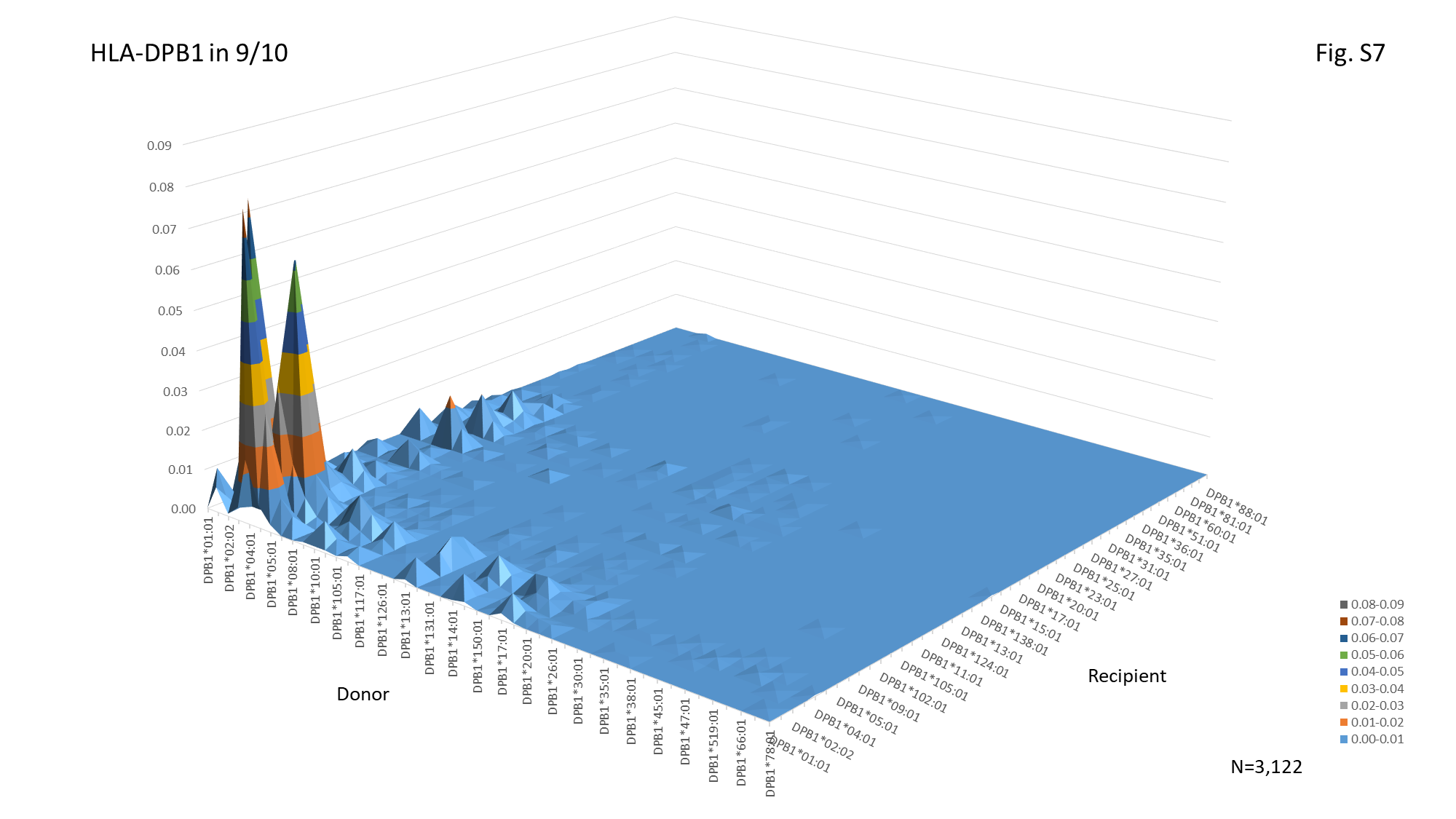


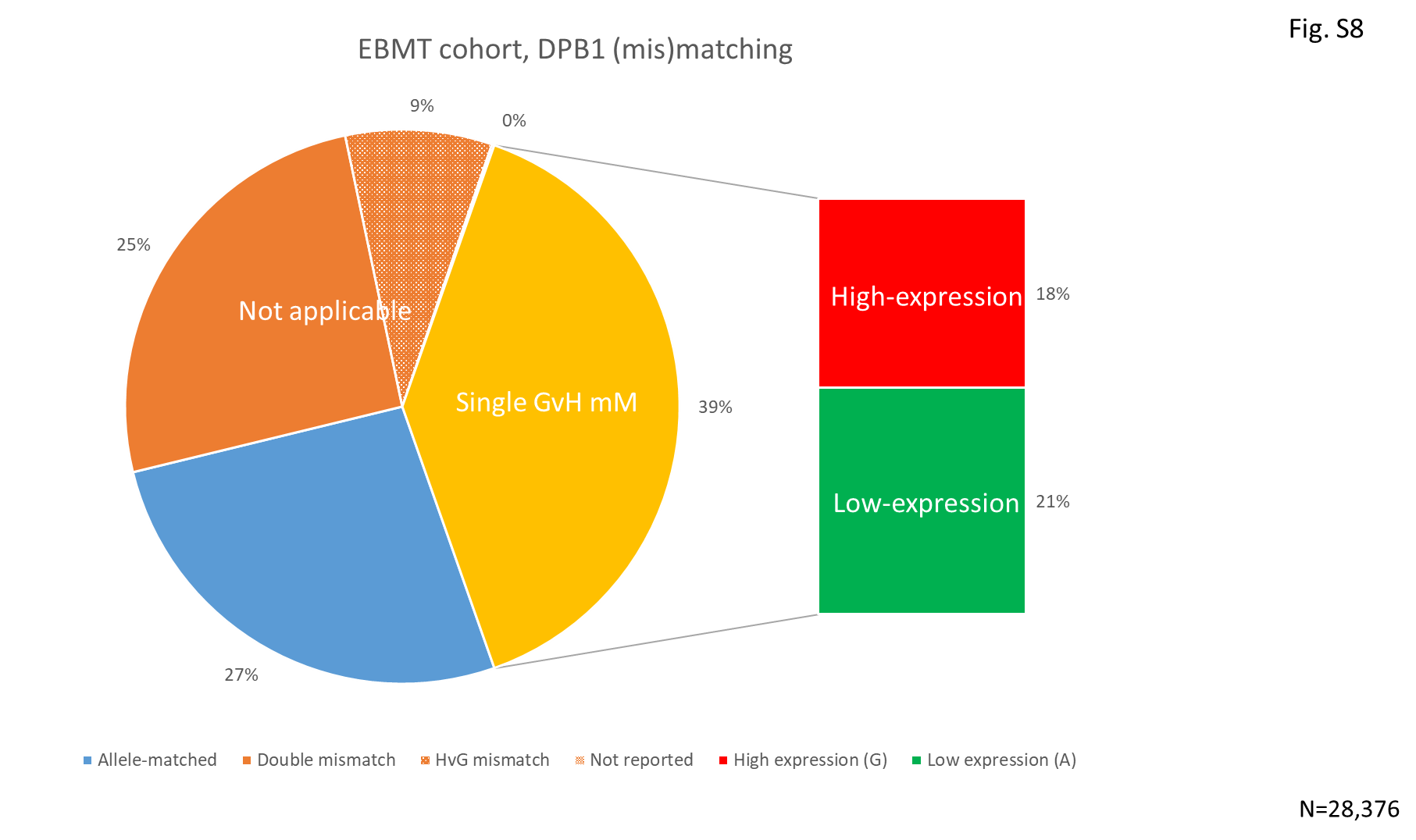


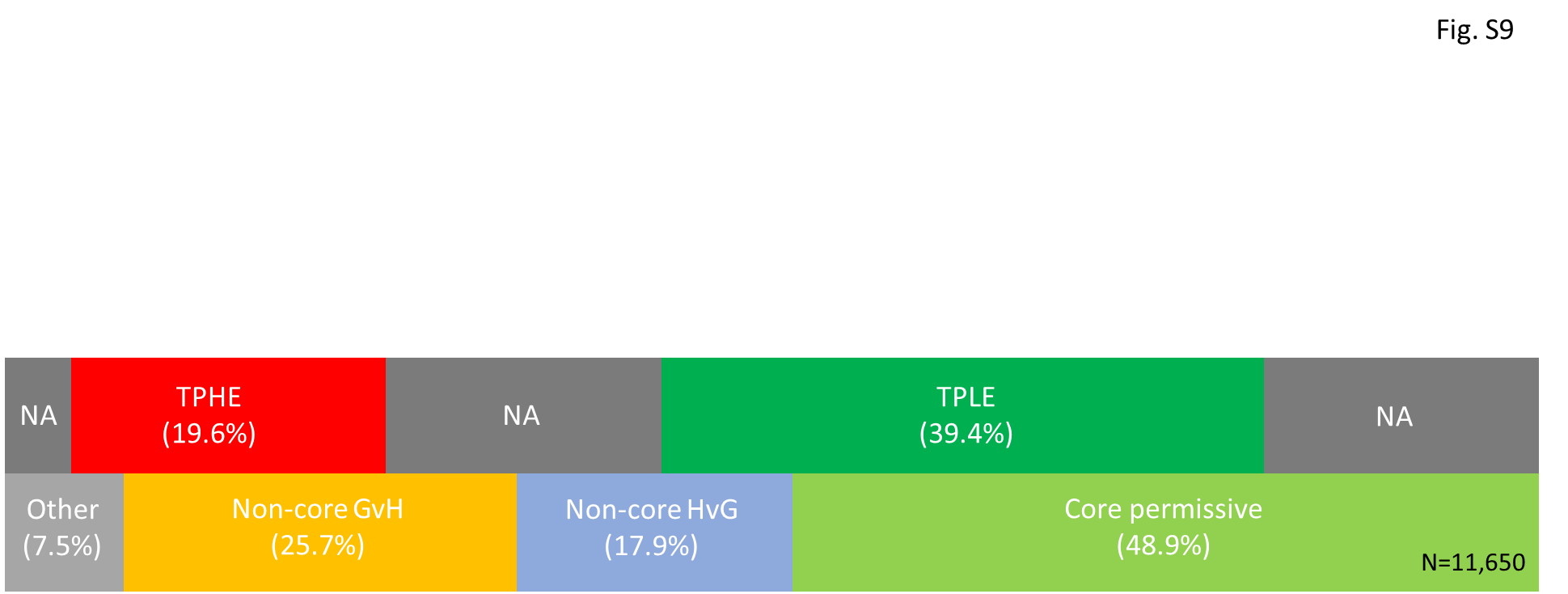

Supplement: Supplementary file 1 — Data S1: tan70637‐sup‐0001‐Supinfo.docx. [file TAN-107-e70637-s001.docx]
